# Research on anti-clogging of ore conveyor belt with static image based on improved Fast-SCNN and U-Net

**DOI:** 10.1038/s41598-023-45186-0

**Published:** 2023-10-19

**Authors:** Jingyi Liu, Hanquan Zhang, Dong Xiao

**Affiliations:** 1 School of Sciences, Northeastern University, Shenyang, 110819 China; 2 School of Information Science and Engineering, Northeastern University, Shenyang, 110819 China

**Keywords:** Engineering, Mechanical engineering

## Abstract

This paper presents an improved Fast-Segmentation Convolutional Neural Network (Fast-SCNN) and U-Net networks based on the channel attention mechanism. While ensuring the speed of network detection, the accuracy of image segmentation is also considered. The experimental results show that the accuracy rate of improved Fast-SCNN based on the channel attention mechanism is greatly improved compared with the original Fast-SCNN, reaching 88.056%, and the mean intersection over union is also improved to a certain extent, reaching 81.087%, and the detection speed is better than the original Fast-SCNN network. The accuracy of improved U-Net network based on the channel attention mechanism is 0.91805, which is better than the original U-Net network. In terms of detection speed, the improved U-Net network based on channel attention mechanism has greatly improved compared with the original U-Net network, reaching 24.02 frames per second. In addition, a method of preventing clogging of ore conveyor belts based on static image detection is proposed in this paper. By judging and predicting the blockage of the ore conveyor belt. When the conveyor belt is about to be blocked or has been blocked, the fuzzy algorithm is used to control the ore conveyor belt to slow down and stop, to improve the safety and efficiency of the conveyor belt.

## Introduction

The mining and smelting of iron ore affect the development level of the iron and steel industry. In the process of ore mining, the use of conveyor belt transportation is of great significance to improve ore mining efficiency and reduce production costs^1^. Problems such as wet ore and large ore particles make it easy to jam in the transportation process, and the production efficiency will be greatly reduced.

The most original conveyor belt blockage is mainly assisted by mechanical devices. In literature^2^, mechanical devices combined with sensors are used to assist in the detection of conveyor belt operation systems, and the monitoring effect is good. However, this method requires that the mechanical device be triggered only when the ore is blocked to a certain extent, and the ore conveyor belt runs at a fast speed, which is prone to false detection during operation. Removing large chunks of ore is also a common method to prevent clogging, but removing large chunks of ore in a conveyor belt running at high speed increases production costs^3^. The screening method^4^ is a relatively simple traditional anti-clogging method, which uses manual or mechanical vibration to make different sizes of ores fall through different screens to prevent large ores from accumulating and blocking the conveyor belt. A similar method is the sedimentation method^5^, which determines the particle size of the ore according to the different velocities of the ore suspension sedimentation, and then separates and selects large ores to avoid blocking the conveyor belt. Ultrasonic detection^6^ is also used to judge the size of the conveyor belt ore and calculate the particle size of the ore by detecting the energy attenuation before and after the ultrasonic wave passes through the interior of the ore. However, this method requires a stricter testing environment, and requires the ore to be degaussed and other operations, which increases the production cost. The maintenance of ultrasonic equipment is also more complex, and each time it needs to be re-calibrated, increasing the complexity of the operation. With time, the emergence of machine learning has brought great convenience to image processing technology^7–9^. Among them, watershed algorithms, genetic algorithms and deep learning are the main methods.

The advantages of the watershed method^10,11^ are fast calculation speed and accurate positioning, which can better extract the ore contour edge. As a result, more and more researchers have applied it to the practice of conveyor belt image segmentation.

Based on the genetic algorithm^12,13^, the main process of the ore conveyor anti-blocking system research is carried out by using the genetic algorithm based on the watershed method. Compared with the traditional particle size detection method, this method can not only save manpower and material costs. At the same time, it can also feedback on the ore size information in real-time to guide the adjustment of the conveyor belt. A camera device is installed above the conveyor belt to capture images of ore particles on the conveyor belt. The image is input to the computer for processing, statistics of different ore sizes, and then distinguish and select large ores to avoid blocking the conveyor belt.

Deep learning is one of the most advanced image processing methods at present, especially semantic segmentation has been more widely used^14^. In recent years, it has been applied in the ore image field. In 2018, Li et al.^15^ used the overall nested edge detection model of the VGG network for segmentation, then processed the segmented image with skeleton thinning, and finally identified the region and marked the color, which could help solve problems such as mutual adhesion and shadow. This method works well for large ores but is less effective for small ores. In 2020, Liu et al.^16^ first extracted the outline of the ore image using U-Net and then used Res-U-Net to perform secondary optimization on the U-Net segmentation results. Experiments show that this method is more accurate than only U-Net segmentation. An improved U-Net structure called Res-U-Net was proposed by Mustafa et al.^17^.The network combines U-Net and residual structure. The high-resolution remote sensing images of iron ore positive, iron ore negative, and background annotation are segmented. The experimental results show that the Res-U-Net method is superior to other image segmentation methods. But the method has never been tried on a conveyor belt. In 2021, Yang et al.^18^ used the VGG16 network as a U-Net encoder to extract ore image features. In addition, the contour sensing loss function is improved, which can give more weight to ore edges and difficult-to-divide areas. Moreover, different levels of features are integrated into the decoder part, which can greatly improve the precision of multi-class ore segmentation. However, the experimental data set is manufactured by artificial staging, which is very different from the actual ore conveyor site. In 2022, Tang et al.^19^ proposed an ore image segmentation method based on Swin-U-Net given the limitations of convolution operations, and better extracted the global and local features of ore images.

Although the traditional anti-blockage method of ore conveyor belt can alleviate the blockage, it has the problems of long time consumption and high cost. In this paper, an improved Fast-SCNN and U-Net image segmentation network based on the channel attention mechanism is proposed. The proposed network can be used for the anti-clog task of the ore conveyor belt and can accurately and quickly segment the images of the ore conveyor belt. The main contributions and originality of this paper are summarized as follows:

1. The ore conveyor belt image segmentation data set is established, including two states of normal operation and blockage, and data enhancement and preprocessing are performed to improve the generalization ability of the network model.
2. Fast-SCNN and U-Net are combined with channel attention mechanism to better extract the features of ore conveyor belt images, to improve the segmentation accuracy and detection speed of the network model.
3. This paper proposes an anti-clogging method for ore conveyor belt based on static image detection. By judging and predicting the blockage of the ore conveyor belt, the fuzzy algorithm is used to control the ore conveyor belt to slow down and stop when the conveyor belt will be blocked or has been blocked.

## Channel attention mechanism

The general idea of the attention mechanism^20^ is to change the focus from the whole to the local important information. Its function is to highlight more important information by suppressing useless information and to focus attention on the information of interest. In principle, it can be divided into three types: spatial attention model, channel attention model, and mixed space and channel attention model. Because not all the information is needed when the image dataset is processed, the main features of the conveyor belt ore must be effectively extracted when performing segmentation. The channel attention mechanism is introduced into the network to help the model focus on more important regions and improve the segmentation accuracy.

**Figure 1:**
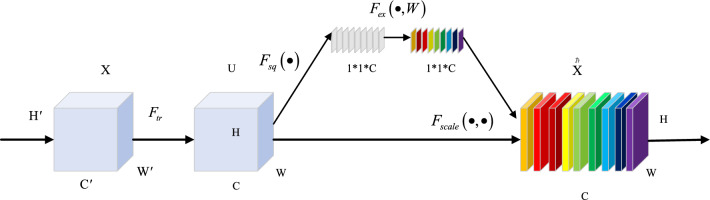
Channel attention mechanism.

The two main steps of the channel attention mechanism^21^ are Squeeze and excitation. The main function of Squeeze is to obtain the Global compressed Feature vector of the current Feature Map by performing Global Average Pooling on the Feature Map layer. The main Excitation function is to obtain the weights of each channel in the Feature Map through the full connection of the two layers. The weighted Feature Map is taken as the input to the next layer network. The main operations are shown in Fig. 1 below.

As shown in the Fig. 1 above, (1) $F_{sq} \left( \bullet \right)$ indicates that Squeeze operation is mainly carried out through global averaging pooling to compress the two-dimensional feature (H*W) of each channel into a real number, and the size of its feature graph changes from (H, W, C) to (1, 1, C). (2) $F_{ex} \left( { \bullet ,W} \right)$ is for the excitation operation. A weight value is generated for each feature channel. The feature map size is still (1, 1, C). (3) $F_{scale} \left( { \bullet , \bullet } \right)$ represents the Scale operation. Through the multiplication operation, the previous normalized weights are superimposed on the features of each channel by weighted operations. The output feature map size changes from (H, W, C) * (1, 1, C) to (H, W, C).

The channel attention mechanism helps to improve the accuracy of image segmentation tasks by adaptively adjusting the weight of feature maps, reducing redundant information, and increasing the importance of key features. It can make the model pay more attention to useful features, and obtain more accurate and detailed segmentation results by expressing image semantics and context information more effectively.

## U-Net network and improved U-Net network

### U-Net network

**Figure 2:**
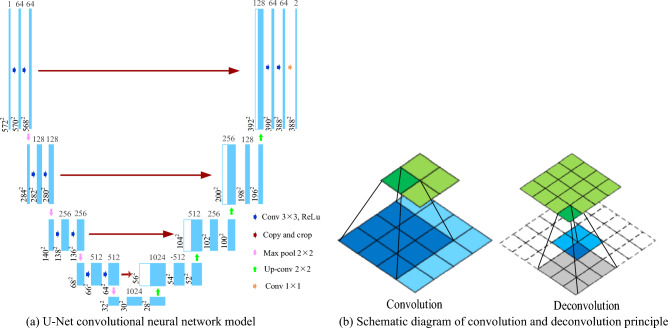
(**a**) U-Net convolutional neural network model; (**b**) Schematic diagram of convolution and deconvolution principle.

U-Net network^22–24^ is named because the overall network has a U-shaped structure, which is obtained by improving based on a fully convolutional neural Network (FCN). The end-to-end segmentation method makes the segmentation results more intuitive. Similarly, the U-Net network is also composed of two main parts: encoding and decoding. U-Net down-sampling is used to extract the features of the data, and up-sampling gives the network a larger field of view. The middle contains skip connections, which are used to combine the bottom layer information with the upper layer information. It was originally proposed for cell segmentation, and then it was widely used in medical image segmentation fields such as fundus blood vessel segmentation. Figure 2a shows the U-Net network structure diagram.

In the U-Net network, the first half of the process is called down-sampling, which mainly includes convolution, pooling, and other operations. In the process of down-sampling, the size of the image is continuously reduced, while the dimension of the data is continuously increased. Up-sampling is the process of enlarging the data so that the resolution of the data is increased. Mainly through row up-sampling operations such as deconvolution and up-pooling. The simplest way to perform up-sampling is through interpolation. Figure 2b shows the operation process of convolution and deconvolution.

U-Net uses a loss function with margin weights:

1 $$E = \sum\limits_{x \in \Omega } {w(x)\log } (p_{l(x)} (x))$$

where $p_{l(x)} (x)$ is the *softmax* loss function, $l:\Omega \to \left\{ {1,....,K} \right\}$ is the label value of the pixel, and $w:\Omega \in R$ is the weight of the pixel in the image.

2 $$w(x) = w_{c} (x) + w_{0} \cdot \exp \left( { - \frac{{\left( {d_{1} (x) + d_{2} (x)} \right)^{2} }}{{2\sigma^{2} }}} \right)$$

U-Net network is one of the early algorithms for the semantic segmentation of people by using multi-scale features. The symmetrical network structure has inspired many subsequent network structures. However, the task of ore conveyor segmentation has great shortcomings, mainly the timeliness of segmentation. Since ore transmission is a process of continuous motion, it is necessary to detect the blockage in time when the blockage occurs at the beginning, to ensure the timidity of subsequent operation and prevent the occurrence of more serious blockage. Given the slow detection of the network, this paper makes some improvements, and the detection speed is greatly improved with less loss of detection accuracy, which ensures the timeliness of the dynamic monitoring process.

### Improved U-Net network

The U-Net algorithm can achieve good segmentation results for general tasks and has great advantages in segmentation accuracy. However, for the segmentation speed, it cannot meet the requirements of real-time segmentation. Due to the fast speed of the ore conveyor belt in operation, once the blockage is not found in time, it will lead to the aggravation of the blockage. Therefore, this paper improves the U-Net algorithm to solve the problem of low detection speed. On the basis of channel reduction for U-Net, the influence of pruning operation on segmentation accuracy is reduced by adding channel attention mechanism. The network greatly improves the detection speed with high segmentation accuracy, which provides a basis for the subsequent network design.

**Figure 3:**
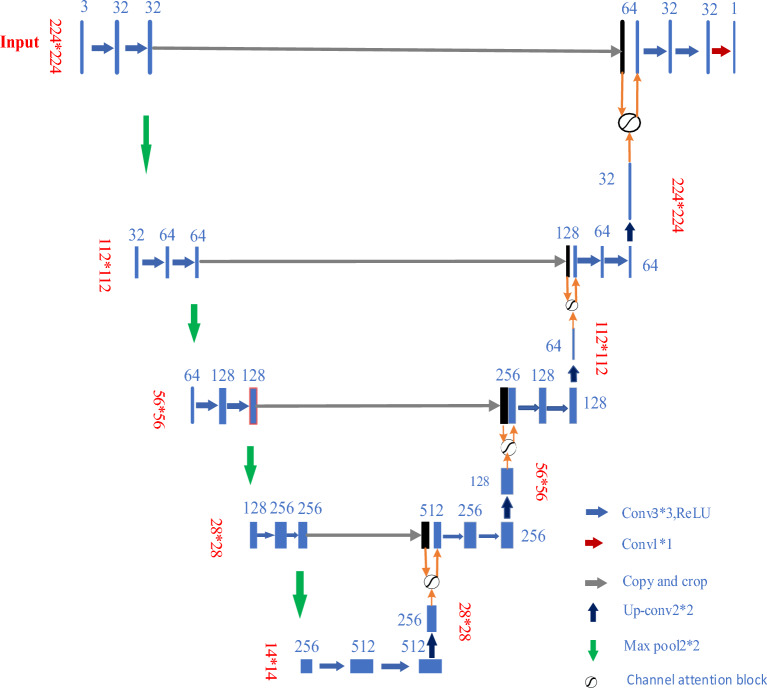
Improved U-Net network with attention mechanism.

As shown in Fig. 3, pair channel reduction in the original U-Net network. The input image is the size of (3, 224, 224), the output is the label map of (1, 224, 224), and the size of the input image is not fixed. At the same time, padding is used in the process of convolution to ensure the consistency of image size. There is a significant drop in accuracy after channel reduction. The paper uses the channel attention mechanism to make up for it, which greatly improves the detection speed of the algorithm.

## Fast-SCNN network and Improved Fast-SCNN network

### Fast-SCNN network

**Figure 4:**
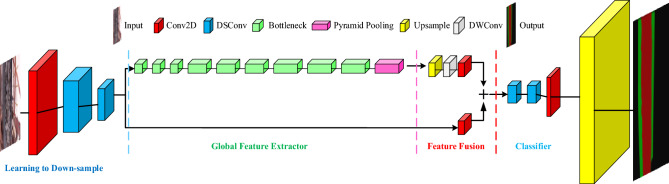
Network structure diagram of Fast-SCNN.

With the advent of automated systems, the timeliness of computation needs to be more urgently considered. Especially with the emergence of autonomous driving technology, it is urgent to improve the real-time performance of network segmentation tasks. From the point of view of the segmentation task on paper, the ore conveyor belt runs at a high speed. Once the blockage cannot be detected in time, it will be more difficult to clean up in a short time and increase the risk of belt damage. It can be seen that timeliness is still a decisive factor to be considered in the segmentation task of this paper, and Encoder-decoder is still the main way of semantic segmentation. The proposal of Fast-SCNN provides high reference significance for such systems with high timeliness requirements. The aforementioned network architecture exhibits a minimal memory footprint, rendering it suitable for deployment on embedded devices. Furthermore, it is capable of delivering real-time effects even when processing high-resolution images. Fast convolutional neural network^25,26^ has a two-branch method based on the existing fast semantic segmentation network, and at the same time introduces a learn to down-sample module, which combines high-resolution spatial information with low-resolution deep features for application. It accelerates the speed of segmentation while maintaining high accuracy, and meets the requirements of timeliness for a segmentation system. On the cityscape’s dataset, it has a speed of 123.5 frames per second and a mean intersection over the union of 68%. Figure 4 shows the network diagram of Fast-SCNN.

It can be concluded from Fig. 4 that the overall structure of the network is still an encoder-decoder structure. Feature extraction is performed through the learning to down-sample as well as the global feature extractor module. The learning to down-sample module uses two depth wise separable convolutions (DS Conv) for feature extraction to improve the speed of operation, and a convolution layer is used before the depth wise separable convolution layer. The global feature extractor is also a feature extraction module, which uses a bottleneck layer followed by a pyramid pooling connection. The feature part uses a quadratic linear interpolation, DW Conv with a convolutional layer for up-sampling. Then, the output result of learning to down-sample is added directly, and the feature is performed again by conv. Finally, the pixel classification is carried out.

### Improved Fast-SCNN network

The two-branch structure adopted by Fast-SCNN introduces a learning down-sampling module to combine high-resolution spatial information with low-resolution deep features. Through parameter sharing and pyramid pooling, the segmentation speed of the algorithm has been greatly improved. When the video is played more than 20 frames per second, it will not feel obvious stagnation, and Fast-SCNN can detect 30 pictures per second, which fully meets the needs of system design. However, the segmentation accuracy is decreased compared with the U-Net network. In order to make up for the lack of segmentation accuracy, the paper improves the basic structure of the algorithm by introducing the attention mechanism. The detection speed of the algorithm is slightly improved compared with the original network so the detection accuracy of the algorithm is further improved.

**Figure 5:**
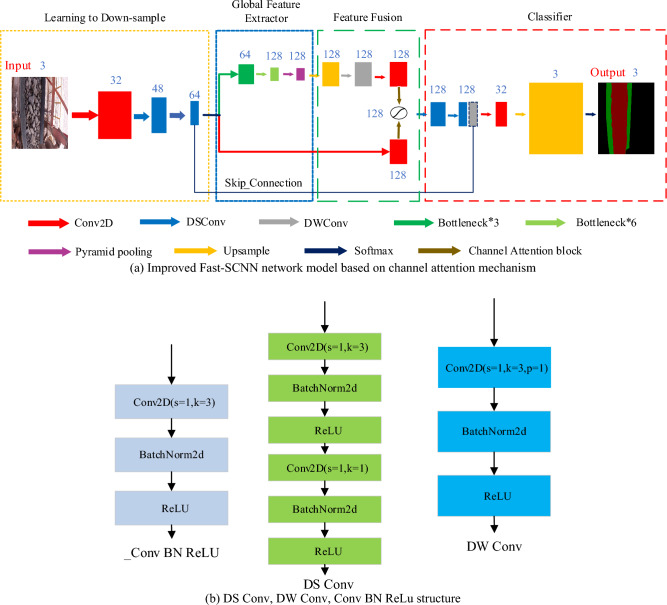
(**a**) Improved Fast-SCNN network model based on channel attention mechanism; (**b**) DS Conv, DW Conv, Conv BN ReLu structure.

In the above network structure, the attention mechanism is used to adjust the network structure in the feature superposition module of the two-branch network, and the output features are sent to the classifier module. *Softmax* is used for classification before feature output, and finally the feature map is output. In the process of building the model, there is no high number of channels to ensure the real-time performance of the operation. At the same time, the output result of the down-sampling part of the model is connected with the output features of the classifier layer and the DS Conv layer by body skip connection, and the feature is carried out, so that the network can retain more high-resolution features. Since the segmentation task in this paper is relatively simple, more kinds of classification tasks are not carried out. Therefore, the channel reduction of the classifier module is carried out, which is more suitable for the classification task of this paper while reducing the network parameters. The detection speed of the algorithm becomes faster and the segmentation accuracy is improved. The algorithm segmentation achieves a more appropriate effect and prepares for the blockage prediction and judgment of the ore conveyor belt. Figure 5a shows the structure diagram of the Fast-SCNN network model improved based on the channel attention mechanism. Table 1 presents the overall architecture of the model.

**Table 1:** Overall architecture of the model.

Learning to down-sample	Global feature extractor	Feature	Classifier
Conv2D(s = 2)	Bottleneck*3(s = 2)	Up sample	DS Conv*2(s = 1)
DSConv(s = 2)	Bottleneck*3(s = 2)	DW Conv.	Conv2D(s = 1)
DSConv(s = 2)	Bottleneck*3(s = 1)	Conv2D(s = 1) Conv2D(s = 1)	Up sample
–	Pyramid pooling	Attention Block	softmax

The network is mainly composed of four modules, in which learning to down-sample is used to down-sample and extract the underlying features. In this part, parameter sharing is used to reduce the number of network parameters and improve the detection speed. Global feature extractor is the part of feature extraction, feature is used for feature, classifier is used for classification, its *s* stands for convolution step, *s* = 2 means step size is 2, *s* = 1 does not change in the convolution process. The number of channels used in the whole network is less, the maximum is 128, and the smaller number of channels also makes the network smaller and maintains a high detection speed. The structures of DS Conv, DW Conv, and _Conv BN ReLu, which constitute pyramid pooling, adopted in the above table, are shown in Fig. 5b below.

## Flowchart of the proposed method

**Figure 6:**
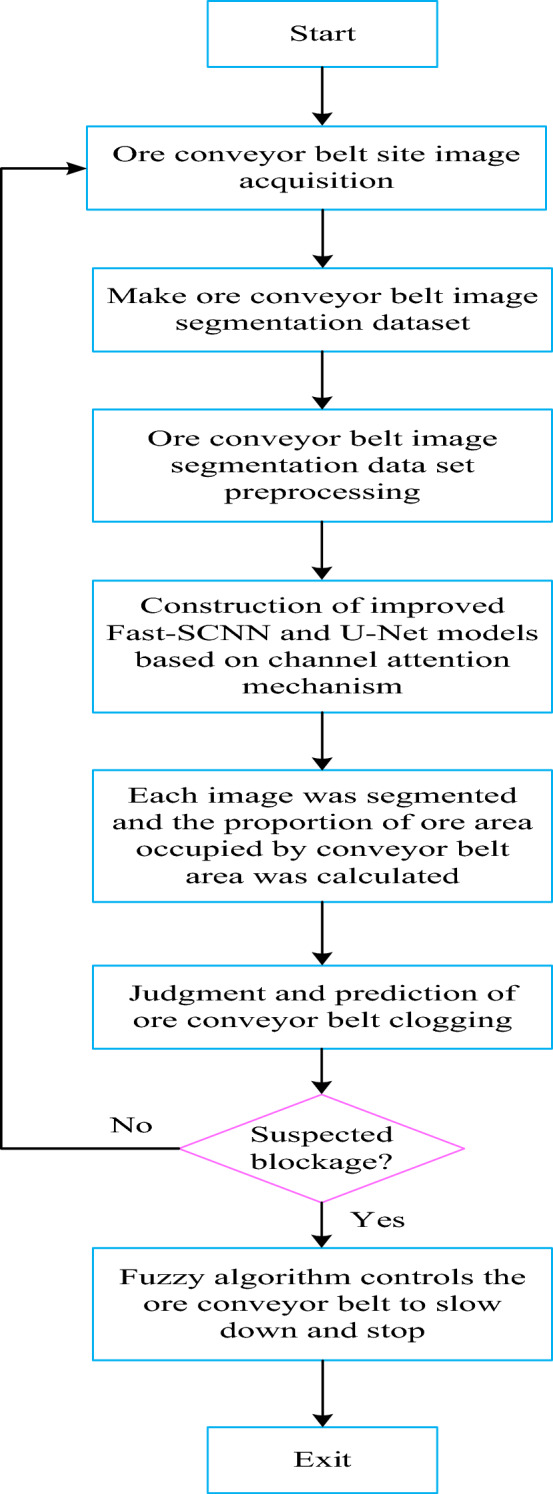
The flow of the proposed method.

The flow of the proposed method is shown in Fig. 6 below. It consists of the following steps and modules: The ore conveyor belt field image acquisition, the production of ore conveyor belt image segmentation dataset, the preprocessing of ore conveyor belt image segmentation dataset, the construction of Fast-SCNN and U-Net models based on channel attention mechanism, the segmentation of each ore conveyor belt image, the calculation of the proportion of ore area occupied by the conveyor belt area, and the judgment of ore conveyor belt blockage Prediction, suspected blockage? and using fuzzy algorithm to control ore conveyor belt to slow down and stop, etc.

## Experimental verification

### Dataset production and processing

In paper, deep learning image segmentation is used to judge the blockage of the conveyor belt. In the case of no suitable public dataset, we choose to build an ore conveyor belt image segmentation dataset by ourselves. The actual data used in this paper are from a large iron mine located in Luliang, Shanxi, China. The data set consists of original pictures and label graphs, each picture corresponds to a label, including a total of 1464 training graphs, 300 test graphs, and 60 verification graphs.

**Figure 7:**
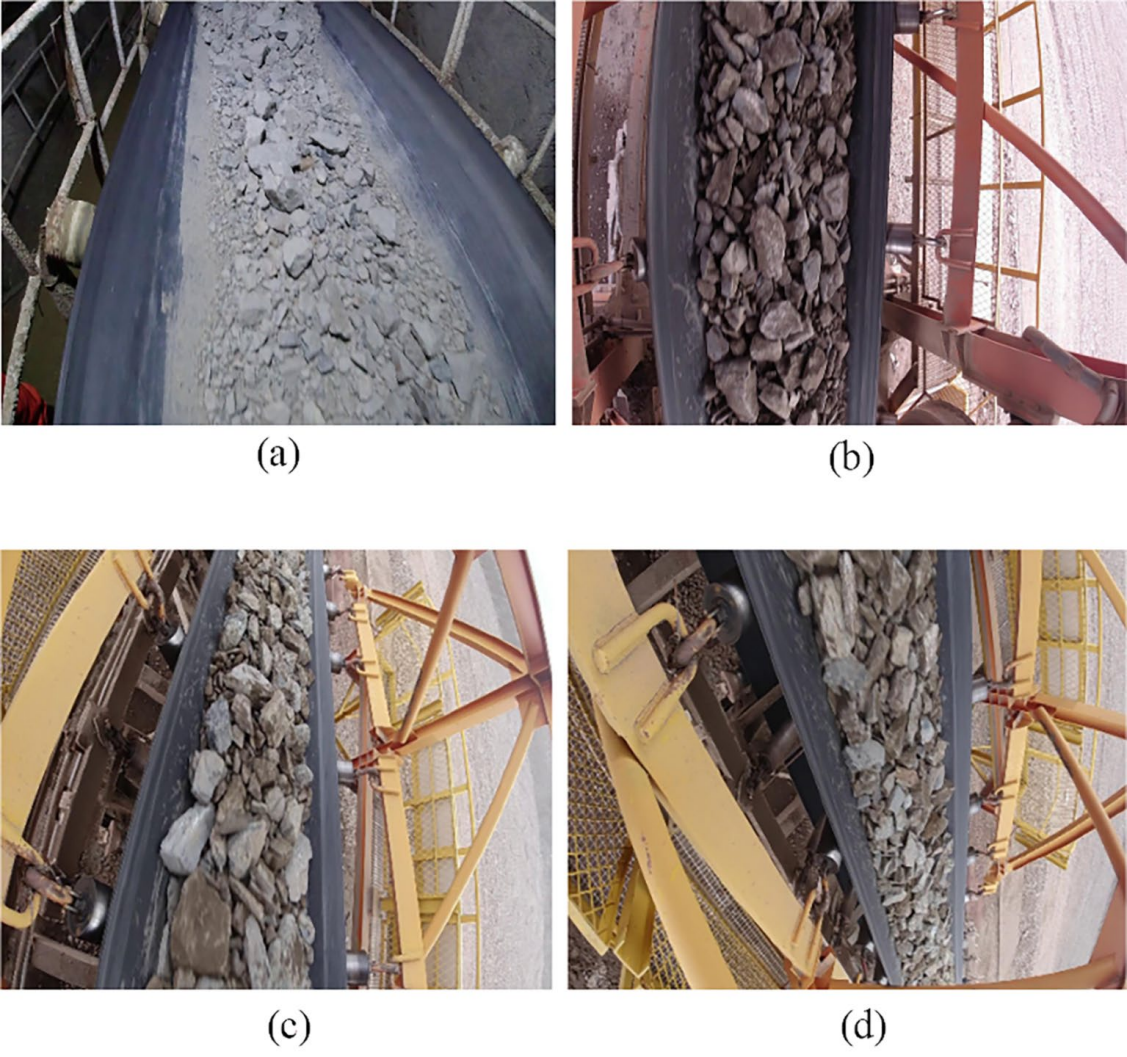
Images taken from different angles.

The images were captured using a variety of equipment, including video from field surveillance cameras, industrial cameras and hand-held cameras. Due to the differences in the frame rate of different devices, there are mainly 24, 120, and 240 frame rate images. Therefore, in order to ensure the accuracy and universality of the network segmentation results, this paper adopts a variety of image processing and data enhancement methods. In the images taken on the spot, a variety of shooting angles and different environments are adopted, and the main angles include several as shown in Fig. 7.

Figure 7 shows several shooting angles of the pictures selected in this paper, among which (a) is the surveillance video inside the mine, which is mainly obtained by tilting the surveillance video to a certain Angle; (b) Images taken in the open air at a vertical Angle; (c) Similar Angle to (a), but the shooting environment is an outdoor open-pit mine; (d) Oblique shooting of an outdoor open-pit mine.

As can be seen from Fig. 7, the operating environment in the field is relatively complex, but the data set captured cannot fully show all the situations that may occur in the field. Therefore, several image transformation methods are used to expand the data set and enrich the data set as much as possible under the premise of ensuring the authenticity of the data. We mainly adopted several methods in Table 2.

**Table 2:** Image handling and problem solving.

Targeted problem	Solution
Simulate shooting position and angle change	The picture rotates and flips
Simulate changes in brightness due to weather and other factors	Limit contrast histogram equalization
Simulate changes caused by noise such as dust	Add multiple noise
Simulate picture sharpness changes	Use interpolation to shrink the image

**Figure 8:**
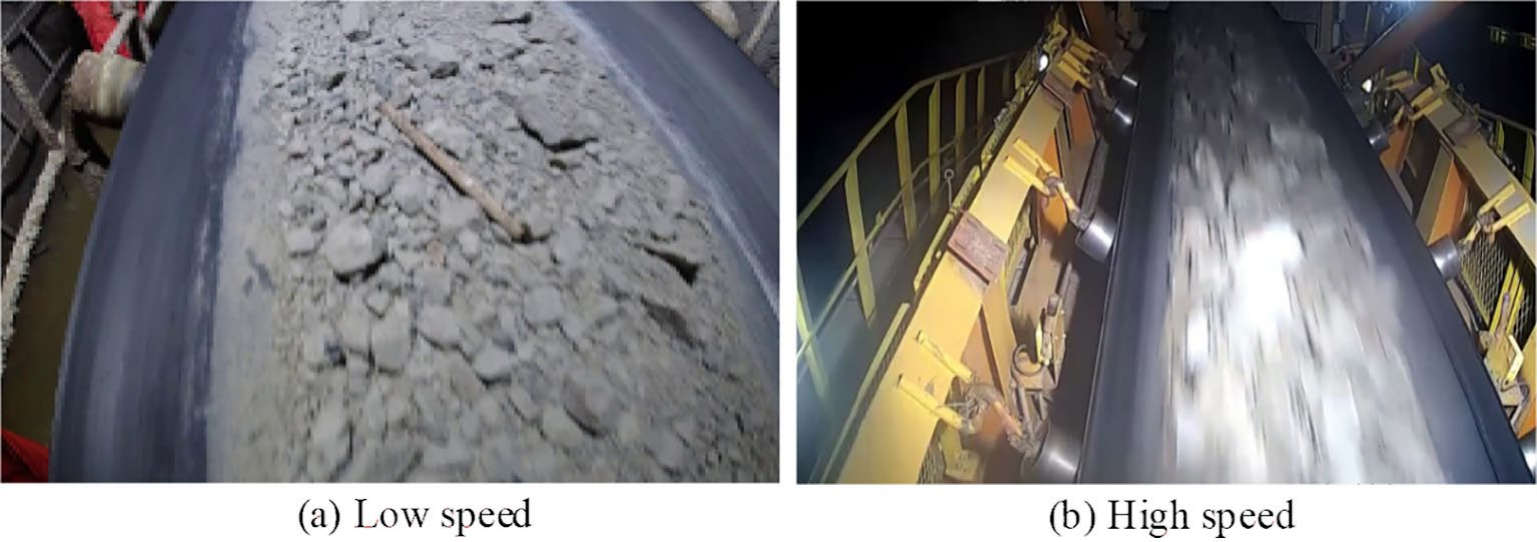
Images of ore conveyor belts at different speeds.

Because of the different speed in the conveyor running process, the different frame rate of the camera has a greater impact on the clarity of the image. Therefore, for some high-speed conveyor belts, high-speed cameras are used to ensure the clarity of the image. In consideration of the segmentation task in this paper, it may need to be used in a faster running environment, and therefore for the integrity of the data set. It is necessary to shoot both the low-speed operation of the conveyor belt and the high-speed operation of the conveyor belt to enrich the integrity of the dataset. Figure 8 below shows the images of the ore conveyor belt at different speeds.

**Figure 9:**
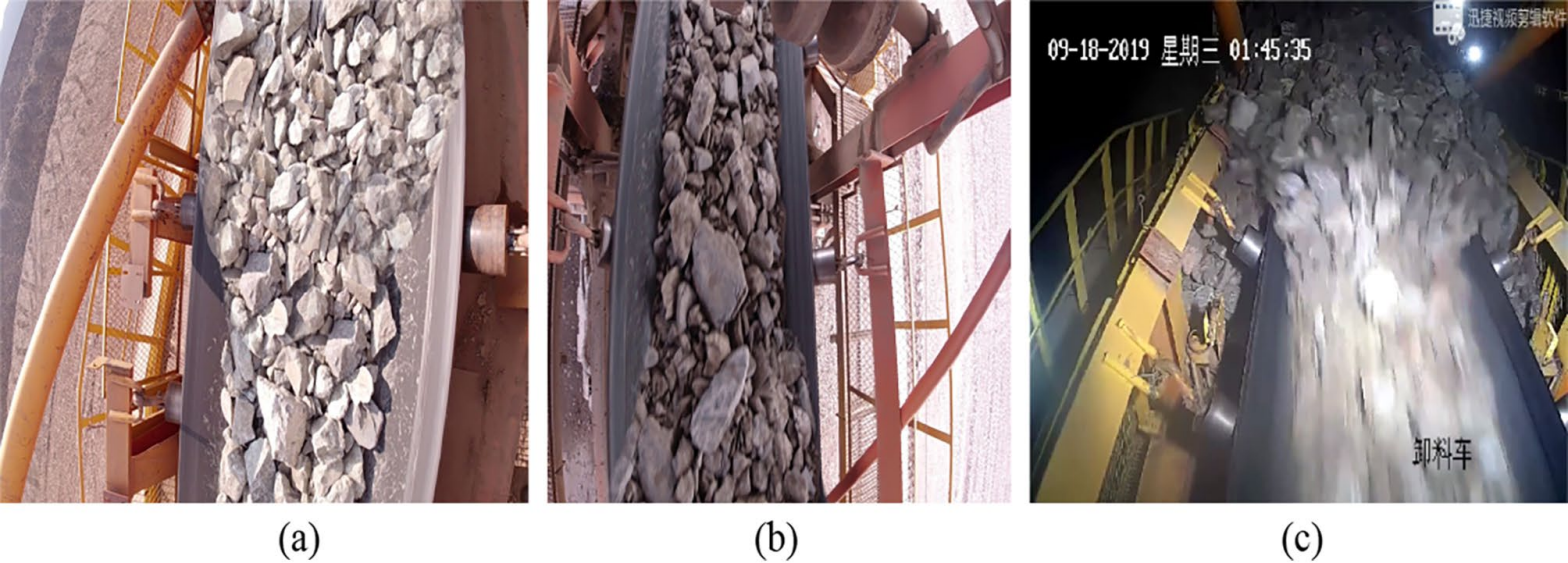
Blockage diagram obtained by treatment (**a**) and (**b**); normal blockage diagram (**c**).

In the process of making the dataset, there are fewer blocked pictures in the dataset collected in the field. In the process of training, to ensure that the training results can better fit the operating conditions of the ore conveyor belt under different conditions, this paper uses photoshop to modify the original pictures. The blockage dataset is expanded by referring to the pictures of normal conveyor belt operation, and the dataset is enhanced for different environmental backgrounds. Figure 9 shows the blocked picture as well as the amplified blocked picture.

### Training setting and evaluation index

The operating system used in the training process of the network model selected in the paper is Windows 10, the graphics card is INTEL(R) CORE(TM) i5-7500 CPU 3.40 GHz processor. The hard drive is a 500 GB solid-state drive and the GPU is an NVIDIA GTX 1050Ti with 32 GB RAM. The programming language is Python 3, the training environment is built by Anaconda software, the main deep learning framework is Pytorch framework, and open cv 4 and PIL are used for image processing. In the process of network construction, Jupyter Notebook was used to build the framework and experiment, and Pycharm software was used to train the network after the network construction was completed. The evaluation metrics used in the training process include accuracy (ACC), mean intersection over union (MIOU), class average pixel accuracy (MPA), and the number of detection frames.

In segmentation tasks, accuracy (ACC) rate is often used as a measure of segmentation, which calculates the proportion of correct pixels in the total pixels in the detection process. ACC is calculated as follows:

3 $${\text{ACC}} = \frac{TP + TN}{{TP + FP + TN + FN}}$$

where *TP*, *TN*, *FP* and *FN* represent true positive samples, true negative samples, false positive samples and false negative samples, respectively.

The average pixel accuracy (MPA) rate is represented as the accuracy statistics of the classified categories, and the calculation method is as follows:

4 $${\text{MPA}} = \sum\limits_{{{\text{i}} = 1}}^{n} {{\text{ACC}}_{i} } /n$$

where *n* represents the number of recognitions, and ACC_*i*_ represents the accuracy rate of this recognition category.

Mean intersection over union (MIOU) is a standard metric for semantic segmentation and is calculated as follows:

5 $${\text{MIOU}} = \frac{1}{k + 1}\sum\limits_{i = 0}^{k} {\frac{TP}{{FP + FN + TP}}}$$

### Experimental results and analysis

**Figure 10:**
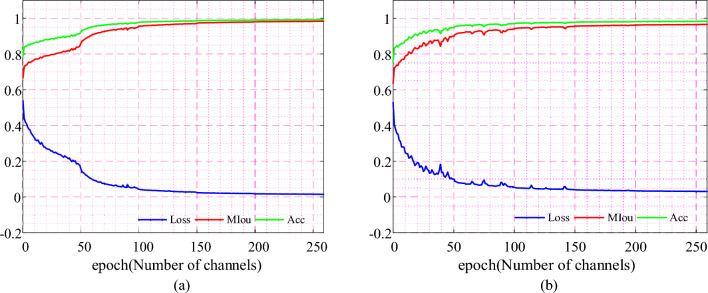
(**a**) Change curve of improved U-Net network training index; (**b**) Change curve of improved Fast-SCNN network training index.

FCN network, SegNet network, U-Net network and its improved network, Fast-SCNN network and its improved network are mainly selected for experimental comparison. In view of the segmentation task in this paper, a more appropriate network is selected for subsequent operations. Figure 10 shows the change curves of accuracy, MIOU and loss function of improved Fast-SCNN and U-Net networks based on the channel attention mechanism during the training process.

It can be seen from Fig. 10a that the improved networks can achieve good training results, but considering the speed of segmentation, the Fast-SCNN network improved based on the attention mechanism is faster. So the Fast-SCNN network adopted subsequently has more advantages than other networks in segmentation speed for ore conveyor belt. However, the segmentation accuracy is lower than that of other networks. The attention mechanism is added during the feature to improve the segmentation accuracy of the network, and the number of channels in the convolutional layer is modified in the classifier layer to make the network smaller and the detection speed is improved compared with the original network. It can be seen from Fig. 10b that 260 epochs were carried out in the training process. In the training process, the loss value was about 0.05, the accuracy reached 0.94, and the MIOU was about 0.95, which tended to be stable afterward. At 260 epochs, the value of the loss function is about 0.04, and the MIOU is about 0.97, which achieves high accuracy.

**Figure 11:**
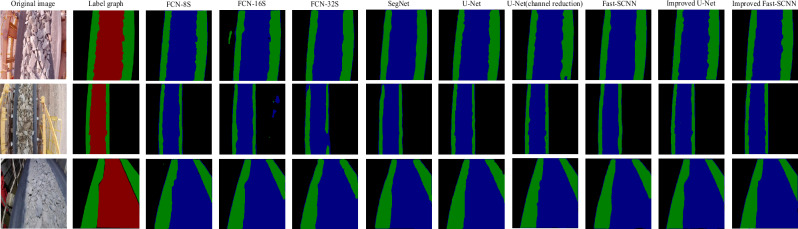
Comparison of segmentation results.

Figure 11 is a comparison diagram of the segmentation experiment results of various methods. It can be seen that the improved Fast-SCNN and U-Net get good segmentation results. The segmentation results of the improved Fast-SCNN and U-Net networks are better than those of the original Fast-SCNN and U-Net networks without improvement respectively. Among all the comparison methods, the improved U-Net segmentation is the best, followed by the improved Fast-SCNN. SegNet segmentation effect is average, FCN-16S and FCN-32S segmentation effect is the worst.

Table 3 shows the evaluation indexes of image segmentation results of various methods. Bold font indicates that this result is optimal. In terms of accuracy, the best is the improved U-Net network reached around 0.92, and the worst is the FCN-8S at 0.88305. In terms of average pixel accuracy (MPA), the FCN-8S performs best at 0.92244 and the FCN-32S performs best at 0.90235. In terms of MIOU, U-Net performed best at 0.82183 and FCN-32S performed worst at 0.77143. In general, most of the indexes of the improved Fast-SCNN and U-Net have been greatly improved compared with the original Fast-SCNN and U-Net .

**Table 3:** Comparison of evaluation indexes of segmentation results.

Network model	ACC	MPA	MIOU	Frames detected per second
FCN-8 s	0.88305	**0.92244**	0.80100	27.12
FCN-16 s	0.86342	0.91324	0.78132	28.31
FCN-32 s	0.84361	0.90235	0.77143	28.65
SegNet	0.90721	0.90532	0.80097	22.03
U-Net	0.91112	0.9049	**0.82183**	14.63
U-Net(channel reduction)	0.90727	0.90793	0.81167	24.95
Fast-SCNN	0.85484	0.90065	0.78751	42.15
Improved U-Net	**0.91805**	0.90370	0.81170	24.02
Improved Fast SCNN	0.88056	0.90341	0.81087	**43.27**

Significant are in value [bold].

The number of frames detected is the number of images that can be divided by detecting test set pictures per second. The accuracy of the original U-Net is high, but the number of frames detected is only 14.63 frames per second. The improved U-Net has a significantly improved detection speed of 24.02 frames per second, which is about 10 frames per second faster than the original U-Net network. The improved Fast-SCNN network can detect 43.27 frames per second, which has the best performance among all comparison methods.

For image segmentation on the low-speed conveyor belt (speed of 2 m/s and below), U-Net incorporating an enhanced channel attention mechanism can be employed, while for the segmentation task on the high-speed conveyor belt (speed of 2 m/s and above), improved Fast-SCNN network integrating the channel attention mechanism can be utilized. The overall segmentation of the ore conveyor belt image is completed. According to different needs, different networks are used to ensure the adaptability of the network for different functions of the conveyor belt.

## Design of ore conveyor belt anti-clogging system based on static image detection

**Figure 12:**
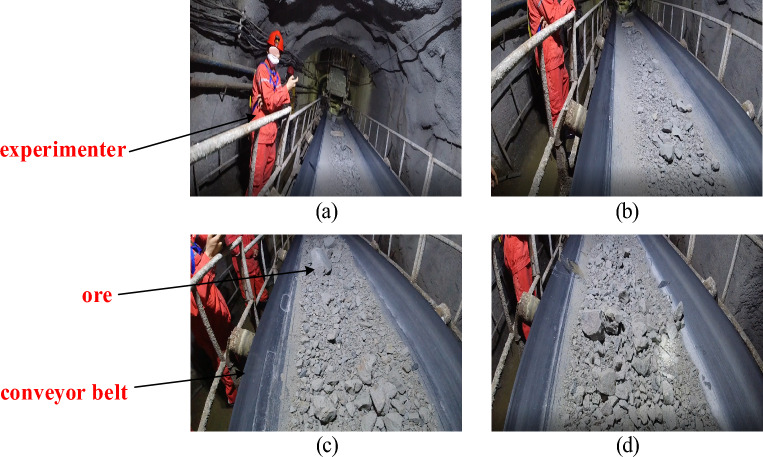
Field diagram of the ore conveyor belt.

According to the experimental analysis in the previous section, by comparing different network segmentation effects, different segmentation methods are used for different speed conveyor belts. In this section, the conveyor belt blockage is judged by static detection image. The running speed of the conveyor belt is controlled according to the different sections of the material proportion, and the speed of the motor is adjusted in time when the material is suspected to be blocked. The on-site running picture of the ore conveyor belt is shown in Fig. 12 below.

The static detection of blockage mainly analyzes the single label picture of the conveyor belt, and judges the blockage in different operating states by comparing the difference in the proportion of materials. The main states include normal, no-load and less material, and blockage state. According to the material coverage ratio of a single picture, the judgment is based on the image statistical map of the data set and the dynamic process map of the blocking material state. The judgment is based on Table 4 below. Figure 9 shows the original image and label image of no-load, normal, blocked and completely blocked by means of static detection.

**Table 4:** Blockage judgment basis and corresponding proportion of materials.

State	Material coverage ratio (%)
No load and less material	0–50
Normal	50–70
Clogging	70–90
Completely clogged up	90–100

**Figure 13:**
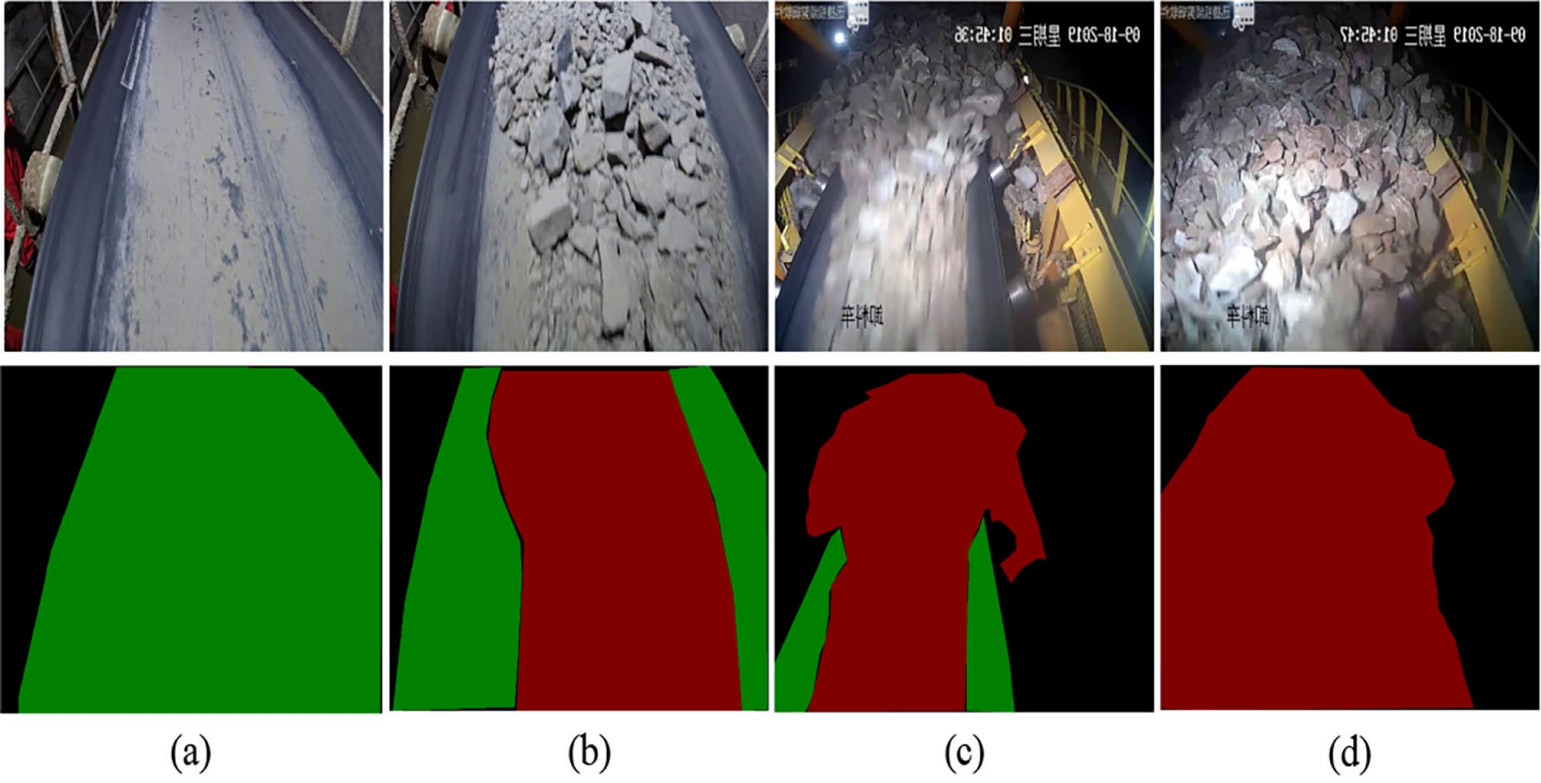
Static detection of different states during the operation of the ore conveyor belt.

It can be seen from Fig. 13 that the ore conveyor belt image has different characteristics under different operation states, (a) is the state of no-load, (b) is the state of normal operation, (c) and (d) are the states of blockage and complete blockage. Especially completely blocked, the conveyor belt has been all occupied by ore, which makes the conveyor belt has been in a state of shutdown. Therefore, using this method to judge the blockage state of the image can quickly detect the blockage state when the blockage occurs. At the same time, the fuzzy algorithm^27–30^ is introduced to control the speed frequency change of the drive motor on the conveyor belt to adjust and change the running speed of the conveyor belt, so that the conveyor belt can maintain a normal and non-blocked state during operation.

In paper, the fuzzy control algorithm combined with the material coverage ratio is used to control the running speed of the belt. According to the actual operation experience, the material proportion of the conveyor belt will continue to change in a relatively stable range. Therefore, according to the continuous change of the material proportion, the material proportion can be associated with the running speed of the conveyor belt, so as to obtain the velocity *v*_prediction_ corresponding to the material proportion, as shown in Table 5 below.

**Table 5:** Material proportion corresponding to velocity values.

Material coverage ratio (%)	Corresponding velocity *v*_*prediction*_ (m/s)
0–50	2.5
50–70	2.5
70–90	0.5
90–100	0

**Figure 14:**
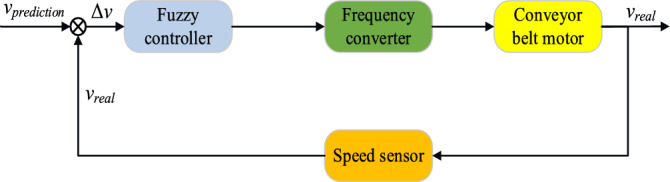
Schematic diagram of the overall control.

The actual running speed of the conveyor belt is obtained according to the speed sensor, and the difference ∆*v* between the real speed value *v*_*real*_ actual and the predicted speed *v*_*prediction*_ is used to control the speed frequency change ∆*f* of the drive motor on the conveyor belt to correct the running speed of the conveyor belt. The different speeds of the conveyor belt operation are optimized by detecting the change of the proportion of materials, and the predicted speed control signal is input into the motor control system. The overall control principle is shown in Fig. 14 below.

According to Table 4, the standard input of the predicted speed of the conveyor belt is carried out, and the error between the real value and the predicted value is obtained by making speed difference with the actual speed signal collected by the speed sensor. Then the fuzzification and defuzzification of the fuzzy algorithm are used to control the change of the speed of the motor, and then the operation speed of the conveyor belt is corrected and optimized, so as to realize the goal of anti-clogging. When there is a tendency to jam, the conveyor belt can be quickly and accurately slowed down, leaving more reaction time for subsequent downtime. Even if blockage occurs, it will not damage the conveyor belt.

## Conclusion

In this paper, an improved Fast-SCNN and U-Net image segmentation network based on channel attention mechanism is proposed. Experiments show that the accuracy rate of improved Fast-SCNN based on channel attention mechanism is greatly improved compared with the original Fast-SCNN, reaching 88.056%, and the MIOU is also improved to a certain extent, reaching 81.087%, and the detection speed is better than that of the original Fast-SCNN network. The accuracy of improved U-Net network based on channel attention mechanism is 0.91805, which is better than the original U-Net network. In terms of detection speed, the improved U-Net network based on the channel attention mechanism has greatly improved compared with the original U-Net network, reaching 24.02 frames per second. In addition, an anti-clogging method of ore conveyor belt based on static image detection is proposed for the first time. When the conveyor belt is going to be blocked or has been blocked, the fuzzy algorithm can be used to control the ore conveyor belt to slow down and stop quickly and accurately, and improve the safety and production efficiency of the conveyor belt.

Although the proposed method can meet the demand of actual conveyor belt production in terms of detection accuracy and detection speed. However, the detection frame number and accuracy of the network still have room for further improvement and whether it can be applied to other kinds of ore conveyor belt needs to be studied in future work.
